# Establishing Reference Data for Fitness Assessment of Law Enforcement Officers Using a Qualitative Systematic Review

**DOI:** 10.3390/healthcare11091253

**Published:** 2023-04-27

**Authors:** Luís Miguel Massuça, Vanessa Santos, Luís Monteiro

**Affiliations:** 1 ICPOL Research Centre, Higher Institute of Police Sciences and Internal Security, 1300-352 Lisbon, Portugal; 2 CIDEFES, Universidade Lusófona, 1749-024 Lisbon, Portugal; 3 CIFI2D, Faculty of Sport, Universidade do Porto, 4200-450 Porto, Portugal; 4 First Responder Research Laboratory, University of Kentucky, Lexington, KY 40506, USA; 5 Exercise and Health Laboratory, Faculdade de Motricidade Humana, Universidade de Lisboa, 1495-751 Cruz Quebrada, Portugal; 6 KinesioLab, Research Unit in Human Movement Analysis, Instituto Piaget, 2805-059 Almada, Portugal

**Keywords:** law enforcement, physical fitness, police officers, normative values for fitness

## Abstract

Physical fitness tests are a standard means of evaluating the competence of police officers. This qualitative review aims (i) to document, compare, and examine the reference values available in the current literature regarding fitness tests for Law Enforcement Officers (LEOs), and (ii) to define reference values for the most used fitness tests to assess and predict police officer performance. A total of 1879 records were collected for review from two major literature databases, PubMed and ScienceDirect. After applying our exclusion criteria, a total of 19 studies were considered. All studies demonstrated acceptable methodological quality in fitness assessment, and the most used components were muscle strength, muscular endurance, muscle power, aerobic and anaerobic capacity, flexibility, and agility. This review provides (i) a methodological definition for the physical fitness assessment that helps select the most used fitness tests, (ii) a standardised methodology for establishing reference data for fitness tests appropriate for LEOs; and (iii) aggregate reference values for selected fitness tests. This may improve selection and retention procedures, considering that this group performs its duties in an environment and under conditions that differ from those of other occupational groups. Complementarily, this qualitative review also provides a foundation for developing effective interventions to improve each aspect of fitness testing for police officers.

## 1. Introduction

In recent years, the demand for emergency services and first responders in public security has increased significantly to protect society from crimes and violence. This has led to a greater emphasis on the physical abilities of officers, highlighting the need for proper fitness testing and training programs.

The profession of Law enforcement officers (LEO) can be physically and mentally demanding. They may be required to perform various physical tasks, such as apprehending subjects, running up and down stairs, pushing their body over obstacles, dragging objects, and engaging in a foot chase. It has been shown that the tasks performed by LEO to protect society from hazards and eliminate threats in real time require adequate physical fitness to be performed efficiently and safely [1,2,3]. Current literature suggests that a large variety of demographic and physical fitness variables are correlated to law enforcement physical ability, including age, body mass index, anaerobic and aerobic capacity, upper-body muscular endurance, lower-body power, and agility [1,2,3].

Many LEO agencies use physical fitness testing as part of the recruitment process to ensure that recruits have the necessary skills to perform academy training [4,5,6]. However, physical fitness also takes on particular importance when results depend on physical fitness performance and promotion processes. Inappropriate assessment protocols for evaluating physical fitness have been reported in concern with LEO [7], providing unclear or limited normative assessment standards [8,9].

Age was associated in several studies as a predictor of a decline in physical fitness [3,9,10]. With increasing age, higher levels of obesity and overweightness, whereas poorer motor skills, have been observed, and LEO are potentially influenced by diseases and risk factors such as hypertension, obesity, diabetes, smoking, dyslipidemia, metabolic syndrome, sedentary lifestyle, and sudden physical and psychological stress [11,12].

Physical fitness testing could be a simple and logical means to motivate police officers to achieve and maintain a minimum level of physical fitness to perform strenuous tasks [13]. Typical fitness programs for LEO often follow a one-size-fits-all approach [14,15]. LEO Campo needs more knowledge and resources with standards or normative values for physical fitness [9,16].

According to Massuça et al. [9], the most commonly used fitness tests to evaluate and predict the performance of police officers were: (i) for muscular endurance, the push-ups, sit-ups, and pull-ups; (ii) for muscular strength, the handgrip and the 1 RM bench press; (iii) for muscular strength, the vertical jump; (iv) for aerobic capacity, the 20-m shuttle run test and the 1.5-mile (2.4-km) run; (v) for agility, the *T*-test; and (vi) for flexibility, the sit and reach.

Therefore, this qualitative review aims: (i) to document, compare, and examine the reference values available in the existing literature related to fitness testing in the LEO; and (ii) to define reference values for the most used fitness tests to evaluate and predict the performance of police officers.

## 2. Materials and Methods

### 2.1. Experimental Approach to the Problem

A review was conducted to identify the reference values for fitness tests used on police officers. This systematic review followed the guidelines of the Preferred Reporting Items for Systematic Reviews and Meta-Analyzes (PRISMA) model [17]. This study is exempt from ethical approval because the authors collected and synthesised data from previous studies in which the investigators had already obtained informed consent. Therefore, this study was not approved by an institutional review board.

### 2.2. Procedures

#### 2.2.1. Search Strategy

To conduct a thorough literature review and obtain relevant original works, we systematically searched major literature databases using specific keywords related to the topic. We searched databases with keywords such as police officer, physical fitness, and health in PubMed (https://pubmed.ncbi.nlm.nih.gov/?term=police+officer+AND+Physical+Fitness+AND+Health&sort=date and ScienceDirect https://www.sciencedirect.com/search?qs=Police%20AND%20Fitness%20test%20AND%20health (accessed on 7 October 2022)) which are known for containing a large number of high-quality, peer-reviewed articles from relevant journals. We summarised the final search terms and applied filters for the databases searched in Table 1.

**Figure 1:**
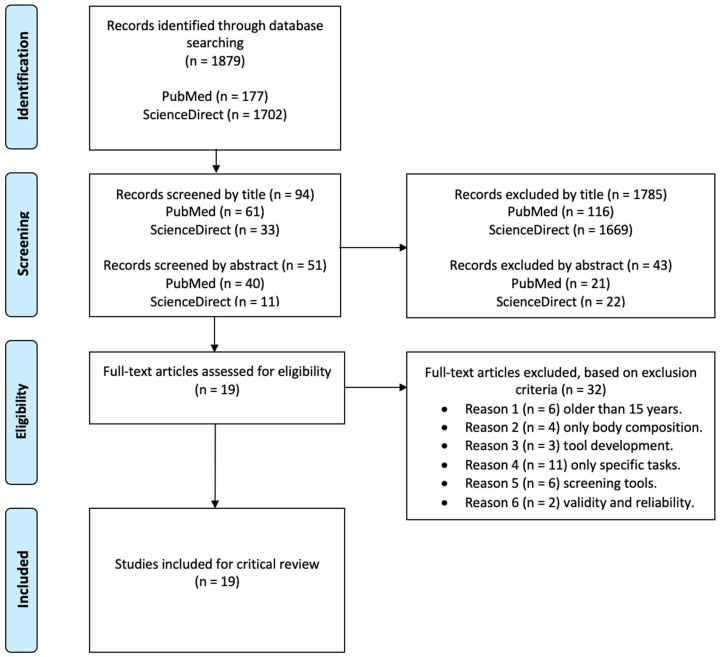
PRISMA diagram detailing the search process.

We aimed to increase the relevance of our search results by applying filters that reflected the study eligibility criteria in each database, where available. These criteria were then used for the full text of articles that passed the initial title and abstract screening process to make a final selection of eligible articles for this qualitative review. The PRISMA flow diagram (Figure 1) [17] documents the search, screening, and selection results. Inclusion criteria were defined as individuals from law enforcement measuring physical fitness and health. In contrast, exclusion criteria were (i) studies older than 15 years, (ii) studies examining only body composition, (iii) studies addressing instrument development, (iv) studies addressing only weight bearing, (v) studies addressing only screening instruments, (vi) validity studies, and (vii) reliability studies. After collecting all studies, duplicates were removed.

#### 2.2.2. Critical Appraisal

We utilised the Critical Appraisal Skill Programme (CASP) checklist, which includes nine questions, to evaluate the study’s methodological quality [18]. Each question had three possible answers: “yes”, “cannot say”, or “no”. As question ten was subjective, we chose to leave it blank. To avoid bias, two authors assessed the methodological quality individually. The results of this quality assessment can be found in Table 2.

#### 2.2.3. Data Extraction

After critical analysis of the full text of the selected articles, a list of intended data was used: (i) authors and year of publication; (ii) study population (country where the study was performed, participants’ gender, age, and intervention groups); (iii) physical capacity evaluated (aerobic capacity; agility; flexibility; muscular endurance; muscular power; muscular strength); and (iv) fitness tests (fitness test results presented as mean ± standard deviation). Table 3 shows data extraction.

#### 2.2.4. Meta-Analysis and Data Aggregation

The data collected from female or male LEO fitness assessment results were subjected to a meta-analysis to establish reference data. We combined the mean estimates and standard deviations of fitness test parameters across several studies. We only aggregated fitness data collected using the same acquisition protocol and collected from the same-sex participants and LEO group (cadets and officers). In accordance, sample size (n), mean estimates (M), and standard deviation (SD) for fitness test results in each of the selected studies were used as effect size estimates. Aggregated effect sizes were calculated using random effect estimating methods (which allows the study outcomes to vary in a normal distribution between studies), i.e., the random effect model was used to compute statistically combined measures and 95% confidence intervals (CI). The restricted maximum likelihood method (REML estimator) was used to estimate the between-sample variance (τ^2^, tau-squared).

The heterogeneity test results should be considered alongside a qualitative assessment of the combinability of studies in a systematic review. To measure the inconsistency of studies’ results, Cochran’s Q (a classical measure of heterogeneity) and the I² (describes the percentage of variation across studies that is due to heterogeneity rather than chance, i.e., expression of the inconsistency of studies’ results; I^2^ = 100% × (Q − df)/Q) were considered [32]. The classification used to evaluate I^2^ is as follows: 0–40%, might not be important heterogeneity; 30–60%, moderate heterogeneity; 50–90%, substantial heterogeneity; 75–100%, considerable heterogeneity (these cut-offs are not absolute, and the interpretation of I^2^ considers the context and clinical relevance of the studies being analysed).

Results of the meta-analysis are also presented in forest plots for matched LEO groups if significant heterogeneity was observed in some fitness tests. Articles that report more than one LEO group of participants within the same sex are written as separate observations in the model. The size of the points on the forest plot is a function of the precision of the outcome, more precise estimates are more prominent in the plot, and their area corresponds to the weight they received in the random effect model. Statistical analysis and forest plots were performed using the Statistical Package for the Social Sciences (IBM Corp. Released 2021. IBM SPSS Statistics for Windows, Version 28.0. Armonk, NY, USA: IBM Corp).

**Table 3:** Data extraction table including fitness tests with their results.

Author/Year of Publication	Study Population	Physical Capacity	Fitness Tests *
Beck et al., 2015 [3]	Law Enforcement Officers USA n = 16 (♂) Age, 33.1 ± 8.7 years	ME	▪ Push-ups (no time limit; reps): 34.8 ± 12.6
		MS	▪ 1 RM bench press (kg): 93.1 ± 19.8 ▪ Handgrip (kg): Left, 52.5 ± 5.9; Right, 55.9 ± 6.4
		MP	▪ Vertical jump (Sargent; cm): 51.4 ± 10.2
		F	▪ Sit-and-reach (cm): 32.1 ± 9.8
		Other ▪ AC: Maximal GXT (mL/kg/min): 42.7 ± 5.9 ▪ Ag: *T*-test (non-traditional *T*-test; s): 18.2 ± 1.6	
Crawley et al., 2016 [11]	Police Cadets Michigan, USA n = 55 (♀, n = 6; ♂, n = 49) Age: ♀, 22.7 ± 2.1 years ♂, 23.4 ± 2.9 years ♀♂, 23 ± 3 years	ME	▪ Push-ups (60 s; reps): ♀, 18 ± 10; ♂, 47 ± 13; ♀♂, 44 ± 15 ▪ Sit-ups (60 s; reps): ♀, 36 ± 8; ♂, 44 ± 8; ♀♂, 43 ± 8
		MS	▪ 1 RM bench press (kg): ♀, 38 ± 8; ♂, 89 ± 27; ♂♀, 85 ± 28 ▪ Handgrip (kg): ♀, Left hand, 31 ± 8; Right hand, 34 ± 5 ♂, Left hand, 52 ± 10; Right hand, 55 ± 10 ♀♂, Left hand, 50 ± 12; Right hand, 53 ± 11
		MP	▪ Vertical jump (Sargent/Abalakov; cm): ♀, 39.9 ± 4.5; ♂, 59.1 ± 11.1; ♀♂, 57.1 ± 12.1
		Ag	▪ *t*-Test (s): ♀, 12.98 ± 1.12; ♂, 11.4 ± 1.2; ♀♂, 11.52 ± 1.52
		F	▪ Sit-and-reach (cm): ♀, 32.1 ± 6.2; ♂, 28.0 ± 8.5; ♀♂, 28.4 ± 8.3
Dawes et al., 2016 [16]	Police Officers Colorado, USA n = 76 (♂) Age: 39.42 ± 8.41 years	ME	▪ Push-ups (60 s; reps): 55.58 ± 17.35 ▪ Sit-ups (60 s; reps): 41.05 ± 6.96
		MS	▪ 1 RM bench press (kg): 93.79 ± 25.91
		MP	▪ Vertical jump (Sargent/Abalakov; cm): 61.26 ± 7.96
		AC	▪ 2.4-km (1.5-mile) run (time; min): 12.75 ± 2.30 ▪ 2.4-km (1.5-mile) run (estimated *V*O_2_max; mL/kg/min): 41.31 ± 6.50
Losty et al., 2016 [19]	Police Officers Trainees Ireland n = 273 (♀, n = 85; ♂, n = 188) Age: ♀♂, 24 ± 4 years	ME	▪ Push-ups (reps): ♀♂, (pre-) 25 ± 17; (post-) 30 ± 19 ▪ Sit-ups (60 s; reps): ♀♂, (pre-) 22 ± 5; (post-) 27 ± 7
		AC	▪ 20-m shuttle run (estimated *V*O_2_max; mL/kg/min): ♀♂, (pre-) 42 ± 8; (post-) 40 ± 7
		F	▪ Sit-and-reach (cm): ♀♂, (pre-) 19 ± 7; (post-) 20 ± 7
Dawes et al., 2017 [10]	Highway Patrol Officers Colorado, USA n = 631 (♀, n = 34; ♂, n = 597) Age: ♀, 36.21 ± 8.45 years ♂, 39.52 ± 8.09 years Age groups (years): (20–29), n = 89 (♀, n = 6; ♂, n = 83) (30–39), n = 218 (♀, n = 16; ♂, n = 202) (40–49), n = 262 (♀, n = 10; ♂, n = 252) (50–59), n = 57 (♀, n = 2; ♂, n = 55) (60–69), n = 5 (♂) [Note: Complementarily, percentile (P5, P10, P15, P20, P25, P30, P35, P40, P45, P50, P55, P60, P65, P70, P75, P80, P85, P90, P95) ranking (push-ups, sit-ups, handgrip, vertical jump, and number of shuttles) were presented for only male police officers.]	ME	▪ Push-ups (60 s; reps): ♀, 20–69 years, 24.24 ± 11.63; 20–29 years, 30.50 ± 9.95; 30–39 years, 25.13 ± 13.05; 40–49 years, 16.83 ± 3.66; 50–59 years, 21.00 ± 15.56 ♂, 20–69 years, 39.09 ± 15.61; 20–29 years, 47.70 ± 14.74; 30–39 years, 40.52 ± 14.96; 40–49 years, 36.70 ± 15.41; 50–59 years, 31.54 ± 14.39; 60–69 years, 39.20 ± 12.68 ♀♂, 20–29 years, 46.52 ± 15.07; 30–39 years, 39.44 ± 15.44; 40–49 years, 36.22 ± 15.53; 50–59 years, 31.15 ± 14.42; 60–69 years, 39.20 ± 12.68 ▪ Sit-ups (60 s; reps): ♀, 20–69 years, 31.06 ± 9.52; 20–29 years, 38.33 ± 10.56; 30–39 years, 28.81 ± 10.51; 40–49 years, 30.78 ± 5.83; 50–59 years, 28.50 ± 2.12 ♂, 20–69 years, 34.46 ± 10.29; 20–29 years, 41.17 ± 8.22; 30–39 years, 36.63 ± 9.67; 40–49 years, 31.73 ± 9.94; 50–59 years, 29.66 ± 9.76; 60–69 years, 25.40 ± 11.89 ♀♂, 20–29 years, 40.98 ± 8.35; 30–39 years, 36.04 ± 9.93; 40–49 years, 31.70 ± 9.82; 50–59 years, 29.62 ± 9.58; 60–69 years, 25.40 ± 11.89
		MS	▪ Handgrip (dominant hand; kg): ♀, 20–69 years, 37.875 ± 5.34; 20–29 years, 37.67 ± 5.57; 30–39 years, 37.20 ± 4.51; 40–49 years, 36.89 ± 5.06; 50–59 years, 48.00 ± 4.24 ♂, 20–69 years, 55.04 ± 7.77; 20–29 years, 54.67 ± 7.47; 30–39 years, 55.97 ± 8.30; 40–49 years, 55.09 ± 7.36; 50–59 years, 52.27 ± 7.76; 60–69 years, 50.20 ± 3.27 ♀♂, 20–29 years, 53.53 ± 8.49; 30–39 years, 54.65 ± 9.40; 40–49 years, 54.46 ± 8.01; 50–59 years, 52.11 ± 7.68; 60–69 years, 50.20 ± 3.27
		MP	▪ Vertical jump (Abalakov; cm): ♀, 20–69 years, 36.80 ± 5.69; 20–29 years, 40.46 ± 8.13; 30–39 years, 36.00 ± 5.82; 40–49 years, 34.95 ± 5.13; 50–59 years, 40.51 ± 10.59 ♂, 20–69 years, 50.74 ± 8.89; 20–29 years, 58.47 ± 8.79; 30–39 years, 52.73 ± 8.03; 40–49 years, 48.29 ± 7.37; 50–59 years, 43.79 ± 8.18; 60–69 years, 40.34 ± 4.39 ♀♂, 20–29 years, 57.25 ± 9.68; 30–39 years, 51.49 ± 9.02; 40–49 years, 47.80 ± 7.70; 50–59 years, 43.66 ± 8.18; 60–69 years, 40.34 ± 4.39
		AC	▪ 20-m shuttle run (number): ♀, 20–69 years, 26.19 ± 10.86; 20–29 years, 33.33 ± 6.41; 30–39 years, 25.93 ± 12.57; 40–49 years, 22.50 ± 10.30; 50–59 years, 21.50 ± 4.95 ♂, 20–69 years, 38.04 ± 19.87; 20–29 years, 55.63 ± 20.90; 30–39 years, 42.19 ± 19.85; 40–49 years, 31.31 ± 15.52; 50–59 years, 26.74 ± 13.20; 60–69 years, 23.40 ± 7.16 ♀♂, 20–29 years, 54.07 ± 21.00; 30–39 years, 40.98 ± 19.84; 40–49 years, 31.01 ± 15.43; 50–59 years, 26.54 ± 13.00; 60–69 years, 23.40 ± 7.16
Violanti et al., 2017 [20]	Police Officers USA n = 1941 (♀, n = 115; ♂, n = 1826) Age: ♀, 33.0 ± 4.8 years ♂, 35.5 ± 6.8 years ♂♀, 35.3 ± 6.7 years Relative body fat (%BF) groups: ♀ (7.0–20.0%BF), n = 37; ♀ (20.2–23.4%BF), n = 39; ♀ (23.7–35.3%BF), n = 39 ♂ (2.7–13.6%BF), n = 601; ♂ (13.8–18.3%BF), n = 621; ♂ (18.4–34.1%BF), n = 604.	ME	▪ Push-ups (60 s; reps): ♀, 7.0–20.0%BF, 39.2 ± 16.3; 20.2–23.4%BF, 32.4 ± 14.0; 23,7–35.3%BF, 27.6 ± 12.0 ♂, 2.7–13.6%BF, 54.7 ± 15.1; 13.8–18.3%BF, 48.1 ± 13.7; 18.4–34.1%BF, 40.6 ± 13.3 ▪ Sit-ups (60 s; reps): ♀, 7.0–20.0%BF, 44.2 ± 9.2; 20.2–23.4%BF, 42.4 ± 8.8; 23.7–35.3%BF, 36.5 ± 9.4 ♂, 2.7–13.6%BF, 46.0 ± 7.9; 13.8–18.3%BF, 42.5 ± 7.8; 18.4–34.1%BF, 38.6 ± 9.8
		AC	▪ 2–4-km (1.5-mile) run (min): ♀, 7.0–20.0%BF, 12.38 ± 1.32; 20.2–23.4%BF, 13.26 ± 1.55; 23.7–35.3%BF, 14.21 ± 2.12 ♂, 2.7–13.6%BF, 11.06 ± 1.27; 13.8–18.3%BF, 12.00 ± 1.37; 18.4–34.1%BF, 13.10 ± 2.13
		F	▪ Sit-and-reach (cm): ♀, 7.0–20.0%BF, 53.3 ± 7.4; 20.2–23.4%BF, 52.6 ± 6.6; 23.7–35.3%BF, 50.8 ± 5.6 ♂, 2.7–13.6%BF, 48.0 ± 7.6; 13.8–18.3%BF, 47.0 ± 7.6; 18.4–34.1%BF, 45.7 ± 7.1
Orr et al., 2018 [21]	Law Enforcement Agency USA n = 164 (♀, n = 25; ♂, n = 139) Police Officers n = 80 (♀, n = 7; ♂, n = 73) Age: ♀, 37.86 ± 3.67 years ♂, 39.43 ± 8.28 years Police Academy Cadets n = 84 (♀, n = 18; ♂, n = 66) Age: ♀, 30.50 ± 5.76 years ♂, 27.96 ± 5.73 years	ME	▪ Push-ups (60 s; reps): ♀, Officers, 32.71 ± 14.04; Cadets, 51.11 ± 12.75 ♂, Officers, 57.76 ±16.42; Cadets, 70.24 ± 12.27 ▪ Sit-ups (60 s; reps): ♀, Officers, 39.86 ± 18.18; Cadets, 46.83 ± 6.82 ♂, Officers, 40.17 ± 7.69; Cadets, 47.29 ± 5.65
		MS	▪ 1 RM bench press (kg): ♀, Officers, 45.45 ± 6.82; Cadets, 57.83 ± 13.93 ♂, Officers, 99.68 ± 21.01; Cadets, 102.65 ± 22.07
		MP	▪ Vertical jump (Abalakov; cm): ♀, Officers, 47.73 ±7.74; Cadets, 46.08 ± 4.70 ♂, Officers, 62.64 ± 6.53; Cadets, 62.84 ± 8.56
		AC	▪ 2.4-km (1.5-mile) run (min): ♀, Officers, 12.82 ± 1.46; Cadets, 12.35 ± 0.82 ♂, Officers 12.73 ± 2.42; Cadets 11.01 ± 1.17
Frio Marins et al., 2019 [22]	Federal Highway Police Officers Brazil n = 13 (♂) Age: 36.8 ± 3.7 years Groups: Unloaded conditions Loaded conditions	MP	▪ Vertical jump (cm): Squat jump (SJ): Unloaded, 29.8 ± 3.5; Loaded, 27.0 ± 3.0 Countermovement jump (CMJ): Unloaded, 36.2 ± 3.8; Loaded, 32.3 ± 3.0 ▪ Standing broad jump (cm): Unloaded, 192.2 ± 13.8; Loaded, 178.2 ± 12.5
		Other AC: Maximal treadmill (*V*O_2_max; mL/kg/min): Unloaded, 46.2 ± 6.6; Loaded, 45.9 ± 7.5	
Kim et al., 2019 [23]	Police Officers Korea n = 372 (♂, n = 334; ♀, n = 38) Age: ♀, 33.9 ± 6.8 years ♂, 41.8 ± 9.0 years Groups: 2014 (♀, n = 24; ♂, n = 295) 2015 (♀, n = 26; ♂, n = 299) 2016 (♀, n = 34; ♂, n = 316) 2017 (♀, n = 36; ♂, n = 315) 2018 (♀, n = 34; ♂, n = 320) 2019 (♀, n = 35; ♂, n = 327)	ME	▪ Push-ups (60 s; reps): ♀ (with knees on the ground), 2014, 42.7 ± 4.3; 2015, 42.3 ± 3.3; 2016, 42.5 ± 3.6; 2017, 41.9 ± 3.1; 2018, 41.4 ± 3.4; 2019, 40.4 ± 2.7 ♂, 2014, 43.1 ± 7.3; 2015, 42.4 ± 6.7; 2016, 43.3 ± 6.5; 2017, 42.8 ± 6.6; 2018, 40.6 ± 6.7; 2019, 38.5 ± 6.0 ▪ Sit-ups (60 s; reps): ♀, 2014, 39.1 ± 6.3; 2015, 39.8 ± 6.5; 2016, 41.8 ± 6.4; 2017, 42.2 ± 6.4; 2018, 40.9 ± 5.7; 2019, 39.9 ± 5.5 ♂, 2014, 46.8 ± 5.6; 2015, 46.4 ± 6.9; 2016, 46.7 ± 5.8; 2017, 46.2 ± 6.5; 2018, 45.6 ± 6.1; 2019, 44.9 ± 6.0
		MS	▪ Handgrip (mean left and right hands; kg): ♀, 2014, 36.4 ± 7.0; 2015, 36.9 ± 6.1; 2016, 36.2 ± 5.6; 2017, 34.9 ± 2.5; 2018, 34.3 ± 3.5; 2019, 34.7 ± 3.4 ♂, 2014, 52.9 ± 6.9; 2015, 53.3 ± 7.2; 2016, 54.1 ± 6.3; 2017, 53.7 ± 5.4; 2018, 53.7 ± 5.6; 2019, 54.4 ± 6.4
Lentz et al., 2019 [24]	Police Officers Canada n = 1006 (♀, n = 146; ♂, n = 860) Age: ♀, 38.4 ± 6.3 years ♂, 40.0 ± 5.7 years ♀♂, 39.7 ± 5.8 years Groups: Uninjured (♀♂, n = 670) Injured (♀♂, n = 336)	ME	▪ Push-ups (reps): ♀♂, Uninjured, 28.7 ± 11.24; Injured, 32,49 ± 10,75 ▪ Pull-ups (reps): ♀♂, Uninjured, 4.45 ± 5.69; Injured, 6.94 ± 5.81
		MS	▪ Handgrip (kg): ♀♂, Left hand, Uninjured, 50.85 ± 10.86; Injured, 49.56 ± 12.19 ♀♂, Right hand, Uninjured, 48.12 ± 10.25; Injured, 51.75 ± 12.31 ♀♂, Left and right hands, Uninjured, 98.97 ± 20.54; Injured, 101.39 ± 24.00
		MP	▪ Vertical jump (inches): ♀♂, Uninjured, 108.92 ± 5.72; Injured, 110.8 ± 6.96
		AC	▪ 20-m shuttle run (estimated *V*O_2_max; mL/kg/min) ♀♂, Uninjured, 42.24 ± 5.86; Injured, 44.02 ± 6.70
Lockie et al., 2019 [25]	Law Enforcement Officers USA n = 383 (♀, n = 21; ♂, n = 362) Age: ♀♂, 38.44 ± 7.40 years ♀, 35.14 ± 5.16 years ♂, 38.64 ± 7.47 years Age groups: ♀ (20–29), 28.50 ± 0.58 years ♀ (30–39), 34.42 ± 2.91 years ♀ (40–49), 42.20 ± 1.30 years ♂ (20–29), 26.80 ± 1.56 years ♂ (30–39), 34.62 ± 3.0 years ♂ (40–49), 43.19 ± 2.57 years ♂ (50–59), 52.55 ± 3.96 years	ME	▪ Push-ups (60 s; reps): ♀, 20–29 years, 31.25 ± 7.85; 30–39 years, 16.25 ± 8.30; 40–49 years, 15.40 ± 7.09 ♂, 20–29 years, 42.89 ± 14.13; 30–39 years, 43.13 ± 14.04; 40–49 years, 40.83 ± 13.30; 50–59 years, 44.10 ± 15.99 ▪ Sit-ups (60 s; reps): ♀, 20–29 years, 28.0 ± 6.78; 30–39 years, 31.83 ± 6.99; 40–49 years, 30.40 ± 5.90 ♂, 20–29 years, 39.56 ±7.56; 30–39 years, 37.47 ± 8.43; 40–49 years, 34.65 ± 8.40; 50–59 years, 33.31 ± 11.72
		MP	▪ Vertical jump (Abalakov; height, cm): ♀, 20–29 years, 37.46 ± 3.36; 30–39 years, 34.40 ± 4.98; 40–49 years, 30.99 ± 6.87 ♂, 20–29 years, 58.89 ± 8.88; 30–39 years, 54.42 ± 8.54; 40–49 years, 50.91 ± 7.23; 50–59 years, 49.44 ± 8.48 ▪ Vertical jump (Abalakov; Power, watts): ♀, 20–29 years, 3505.64 ± 920.81; 30–39 years, 3493.37 ± 651.13; 40–49 years, 3584.96 ± 961.15 ♂, 20–29 years, 5548.13 ± 795.37; 30–39 years, 5393.54 ± 920.49; 40–49 years, 5280.08 ± 814.42; 50–59 years, 4764.89 ± 1116.63
		AC	▪ 2.4-km (1.5-mile) run (min): ♀, 20–29 years, 15.19 ± 2.16; 30–39 years, 18.08 ± 2.16; 40–49 years, 19.04 ± 3.13 ♂, 20–29 years, 13.31 ± 2.41; 30–39 years, 14.29 ± 3.07; 40–49 years, 15.30 ± 2.56; 50–59 years, 15.29 ± 2.12
		F	▪ Sit-and-reach (cm): ♀, 20–29 years, 54.45 ± 3.82; 30–39 years, 49.16 ± 8.55; 40–49 years, 53.34 ± 8.81 ♂, 20–29 years, 44.65 ± 8.34; 30–39 years, 45.50 ± 7.51; 40–49 years, 46.36 ± 7.16; 50–59 years, 46.78 ± 7.47
Myers et al., 2019 [26]	Law Enforcement Officers USA n = 398 (♀, n = 11; ♂, n = 387) Law Enforcement Agencies: LEA1, n = 79 (♀, n = 7; ♂, n = 72) LEA2, n = 319 (♀, n = 4; ♂, n = 315) Age (groups): ♀ (LEA1), 38.14 ± 3.84 years ♂ (LEA1), 39.43 ± 8.28 years ♀ (LEA2), 32.0 ± 7.07 years ♂ (LEA2), 37.9 ± 7.71 years	ME	▪ Push-ups (60 s; reps): ♂, LEA1, 57.76 ± 16.42; LEA2, 42.16 ± 13.59 ♀♂, LEA1, 55.69 ± 17.33; LEA2, 41.96 ± 13.77 ▪ Sit-ups (60 s; reps): ♂, LEA1, 40.16 ± 8.00; LEA2, 36.96 ± 6.53 ♀♂, LEA1, 40.64 ± 7.63; LEA2, 36.9 ± 8.0
		MP	▪ Vertical Jump (Abalakov; cm): ♂, LEA1, 62.63 ± 6.53; LEA2, 53.06 ± 7.77 ♀♂, LEA1, 61.53 ± 7.30; LEA2, 52.81 ± 8.05
		AC	▪ 2.4-km (1.5-mile) run (estimated *V*O_2_max, mL/kg/min): ♂, LEA1, 41.44 ± 6.81 ♀♂, LEA1, 41.52 ± 6.54 ▪ 20-m shuttle run (estimated *V*O_2_max, mL/kg/min): ♂, LEA2, 34.1 ± 5.51 ♀♂, LEA2, 34.03 ± 5.51
Teixeira et al., 2019 [27]	Police Officers Portugal n = 97 (♂) Age categories (years): 20–29 (n = 43; age, 25.19 ± 2.65 yrs) 30–39 (n = 24; age, 33.29 ± 2.77 yrs) 40–49 (n = 20; age, 44.65 ± 3.18 yrs) > 49 (n = 10; 52.30 ± 2.26 yrs)	ME	▪ Push-ups (60 s; reps): 20–29 years, 56.02 ± 16.70; 30–39 years, 38.88 ± 12.93; 40–49 years, 31.35 ± 15.99; >49 years, 18.70 ± 8.99 ▪ Sit-ups (60 s; reps): 20–29 years, 51.35 ± 8.46; 30–39 years, 37.79 ± 9.08; 40–49 years, 30.10 ± 11.66; >49 years, 24.10 ± 5.82
		MS	▪ 1 RM bench press (kg): 20–29 years, 95.62 ± 17.82; 30–39 years, 83.10 ± 18.36; 40–49 years, 84.7 ± 29.89; >49 years, 64.00 ± 7.02 ▪ Handgrip (left and right hands; kg): 20–29 years, 114.34 ± 12.04; 30–39 years, 104.79 ± 13.47; 40–49 years, 106.63 ± 15.12; >49 years, 100.58 ± 13.02
		MP	▪ Vertical jump (Countermovement jump-CMJ) Height (cm): 20–29 years, 32.02 ± 5.38; 30–39 years, 27.79 ± 6.27; 40–49 years, 24.01 ± 5.46; >49 years, 20.48 ± 5.85 P_max_ (W): 20–29 years, 3456.62 ± 409.21; 30–39 years, 3277.09 ± 419.52; 40–49 years, 3186.01 ± 688.25; >49 years, 2827.54 ± 646.28 ▪ Standing broad jump (m): 20–29 years, 222 ± 15; 30–39 years, 208 ± 11; 40–49 years, 195 ± 17; >49 years, 169 ± 23
		Other evaluation AC: Jackson non-exercise [33] (estimated *V*O_2_max; mL/kg/min): 20–29 years, 48.94 ± 3.46; 30–39 years, 45.94 ± 4.18; 40–49 years, 37.10 ± 6.04; >49 years, 34.30 ± 4.33	
Kukić et al., 2020 [14]	Police Students Serbia n = 177 (♀, n = 79; ♂, n = 98) Age: ♀, 20.9 ± 1.4 years ♂, 20.6 ± 1.3 years	ME	▪ Sit-ups (30 s; reps): ♀, 22.99 ± 2.05; ♂, 26.18 ± 2.71; ♀♂, 24.76 ± 2.91
		MS	▪ Handgrip (daN): ♀, 39.03 ± 4.26; ♂, 63.19 ± 7.24; ♀♂, 52.41 ± 13.49
		MP	▪ Standing broad jump (cm): ♀, 182.08 ± 14.63; ♂, 233.32 ± 15.98; ♀♂, 210.45 ± 29.80
		AC	▪ Cooper (12-min run; m): ♀, 2168.48 ± 193.52; ♂, 2731.43 ± 171.89; ♀♂, 2480.17 ± 334.13
Lockie et al., 2020 [15]	Law Enforcement Agency-Recruits USA n = 908 (♀, n = 147; ♂, n = 761) Age: ♀, 26.97 ± 4.78 years ♂, 27.19 ± 5.86 years ♂♀, 27.16 ± 5.70 years Class number (♂♀): 1 (n = 90; age, 26.87 ± 5.27 years) 2 (n = 93; age, 28.12 ± 6.12 years) 3 (n = 66; age, 25.77 ± 4.06 years) 4 (n = 79; age, 27.22 ± 6.20 years) 5 (n = 67; age, 26.58 ± 5.66 years) 6 (n = 88; age, 27.14 ± 5.63 years) 7 (n = 83; age, 26.88 ± 5.05 years) 8 (n = 84; age, 27.92 ± 6.57 years) 9 (n = 79; age, 27.04 ± 5.25 years) 10 (n = 89; age, 26.92 ± 6.15 years) 11 (n = 88; age, 27.68 ± 5.86 years)	ME	▪ Push-ups (120 s; reps): ♀♂, Class 1, 46.84 ± 7.20; Class 2, 48.16 ± 15.09; Class 3, 47.19 ± 13.5; Class 4, 43.76 ± 13.69; Class 5, 47.16 ± 6.18; Class 6, 50.94 ± 19.20; Class 7, 41.59 ± 11.83; Class 8, 44.52 ± 10.17; Class 9, 48.34 ± 4.43; Class 10, 47.56 ± 12.98; Class 11, 45.06 ± 13.67 ♀♂, Percentile rank: P0-P9, ≤30; P10-P19, 31–37; P20-P29, 38–42; P30-P39, 43–49; P40-P79, 50; P80-P89, 51–58; P90-P100, ≥59 ▪ Sit-ups (120 s; reps): ♀♂, Class 1, 53.50 ± 14.47; Class 2, 54.16 ± 13.69; Class 3, 56.09 ± 16.85; Class 4, 60.20 ± 14.71; Class 5, 60.15 ± 12.51; Class 6, 56.25 ± 16.95; Class 7, 53.27 ± 15.24; Class 8, 55.95 ± 13.53; Class 9, 59.48 ± 13.73; Class 10, 54.98 ± 14.27; Class 11, 47.97 ± 13.59 ♀♂, Percentile rank: P0-P9, ≤36; P10-P19, 37–41; P20-P29, 42–47; P30-P39, 48–52; P40-P49, 53–55; P50-P59, 56–60; P60-P69, 61–64; P70-P79, 65–69; P80-P89, 70–75; P90-P100, ≥76 ▪ Pull-ups (reps): ♀♂, Class 1, 10.16 ± 6.60; Class 2, 7.87 ± 4.90; Class 3, 9.22 ± 5.89; Class 4, 9.03 ± 6.03; Class 5, 11.25 ± 7.26; Class 6, 8.98 ± 7.45; Class 7, 8.16 ± 7.09; Class 8, 9.04 ± 6.74; Class 9, 9.84 ± 5.91; Class 10, 8.69 ± 6.54; Class 11, 8.34 ± 6.74 ♀♂, Percentile rank: P0-P12, 0; P13-P19, 1–2; P20-P29, 3–5; P30-P39, 6–7; P40-P49, 8–9; P50-P59, 10; P60-P69, 11–12; P70-P79, 13–15; P80-P89, 16–20; P90-P100, ≥21
		AC	▪ 2.4-km (1.5-mile) run (min): ♀♂, Class 1, 12.01 ± 1.10; Class 2, 11.58 ± 1.15; Class 3, 12.34 ± 1.35; Class 4, 12.25 ± 1.24; Class 5, 11.10 ± 0.59; Class 6, 12.32 ± 1.21; Class 7, 12.29 ± 1.16; Class 8, 11.51 ± 1.46; Class 9, 11.02 ± 1.01; Class 10, 12.15 ± 1.17; Class 11, 12.53 ± 1.54 ♀♂, Percentile rank: P0-P9, ≥14.02; P10-P19, 13.15–14.01; P20-P29, 12.47–13.14; P30-P39, 12.26–12.46; P40-P49, 12.05–12.25; P50-P59, 11.49–12.05; P60-P69, 11.24–11.48; P70-P79, 10.56–11.23; P80-P89, 10.20–10.55; P90-P100, 7.50–10.19
Araújo et al., 2021 [28]	Police Officers (Special Police Unit) Portugal n = 117 (♂) Age, 42.5 ± 4.4 years	ME	▪ Push-ups (60 s; reps): 49.3 ± 12.2 ▪ Sit-ups (120 s; reps): 62.8 ± 12.5 ▪ Pull-ups (60 s; reps): 10.7 ± 4.9
		MS	▪ 1 RM bench press (kg): 93.0 ± 18.6 ▪ Handgrip (kg): Left hand, 51.7 ± 7.1; Right hand, 53.9 ± 7.6
		MP	▪ Vertical jump (Squat jump–SJ; cm): 31.0 ± 4.8 ▪ Medicine ball throw (3-kg; m): 5.4 ± 0.72
		AC	▪ Cooper (12-min run–distance; m): 2747.5 ± 254.5 ▪ Cooper (12-min run; estimated *V*O_2_max, mL/kg/min): 50.1 ± 5.7
		F	▪ Sit-and-reach (cm): 30.7 ± 7.6
Caetano et al., 2021 [29]	Military Police Paraná, Brazil n = 1705 (♀♂) Year groups: 2016 (n = 103) 2017 (n = 664) 2018 (n = 410) 2019 (n = 528)	ME	▪ Upper body strength (pull-ups, flexed-arm hang, or push-ups)-Unclear. ♀♂, 2016, 42.34 ± 32.55; 2017, 60.33 ± 28.55; 2018, 60.07 ± 28.38; 2019, 57.83 ± 28.53
		AC	▪ 20-m shuttle run (number): ♀♂, 2016, 88.29 ± 20.75; 2017, 95.64 ± 11.02; 2018, 96.56 ± 9.01; 2019, 96.91 ± 7.54 ▪ 12-min run-Cooper (estimated *V*O_2_max): ♀♂, 2016, 42.34 ± 32.55; 2017, 60.33 ± 28.55; 2018, 60.07 ± 28.38; 2019, 57.83 ± 28.53
Lockie et al., 2021 [30]	Law Enforcement Agency Recruits USA n = 514 (♀♂) Graduate (GRAD, n = 436) Age: ♀, 26.7 ± 5.0 years ♂, 26.6 ± 5.3 years ♂♀, 26.6 ± 5.3 years Separate (SEP, n = 78) Age: ♀, 30.5 ± 12.0 years ♂, 32.3 ± 9.2 years ♂♀, 31.8 ± 10.1 years	ME	▪ Push-ups (60 s; reps): ♀♂, GRAD Hiring: 40.32 ± 14.25 ♀♂, GRAD Academy: 42.96 ± 14.77 ♀♂, SEP Hiring: 33.24 ± 11.88 ♀♂, SEP Academy: 35.36 ± 13.43 ▪ Sit-ups (60 s; reps): ♀♂, GRAD Hiring: 39.94 ± 9.15 ♀♂, GRAD Academy: 32.75 ± 13.17 ♀♂, SEP Hiring: 37.55 ± 7.83 ♀♂, SEP Academy: 30.23 ± 11.53
		AC	▪ 2.4-km (1.5-mile) run (min): ♀♂, GRAD Hiring: 12.49 ± 1.32 ♀♂, GRAD Academy: 11.55 ± 1.25 ♀♂, SEP Hiring: 13.44 ± 1.27 ♀♂, SEP Academy: 13.17 ± 1.12
Sá et al., 2021 [31]	Police Officers Close Protection Unit-recruits Portugal n = 32 (♀♂; Age, 30.1 ± 2.7 years)	ME	▪ Push-ups (90 s; reps): ♀♂, 65.4 ± 17.3 ▪ Sit-ups (120 s; reps): ♀♂, 76.9 ± 11.6 ▪ Pull-ups (120 s; reps): ♀♂, 16.5 ± 3.0
		AC	▪ Cooper (12-min run–distance; m), ♀♂, 2729.6 ± 209.0 ▪ Cooper (12-min run; estimated VO2max, mL/kg/min), ♀♂, 49.6 ± 4.7

Key: *, fitness test results presented as mean ± standard deviation (SD);♀, female; ♀♂, female and male; ♂, male;%BF, relative body fat; AC, Aerobic capacity; Ag, Agility; daN, Decanewton (1 daN = 1.0197162129779 kgf); F, Flexibility; LEA, Law Enforcement Agency; ME, Muscular Endurance; MP, Muscular Power; MS, Muscular Strength; reps, repetitions; s, seconds; USA, United States of America; *V*O_2_max, maximum rate of oxygen consumption.

## 3. Results

### 3.1. Search Results

A total of 1879 studies were found during the initial search of the two databases. After removing duplicates and screening by title and abstract, the full-text versions of 51 studies were compiled for review. These studies were then assessed against the inclusion and exclusion criteria, leaving 19 studies for critical review (Table 3). A summary of the screening and selection process and the literature search results can be found in the PRISMA flow diagram [17] (Figure 1). Of the 19 studies, three referred to Portuguese police officers, and the other seventeen referred to police officers from around the world (Brazil, Canada, Germany, Ireland, Korea, Serbia, and the USA). Fifteen studies examined male and female participants, while four included only male participants. The average age of the studies is 34.59 ± 5.58 years old.

### 3.2. Fitness Measures

**Figure 2:**
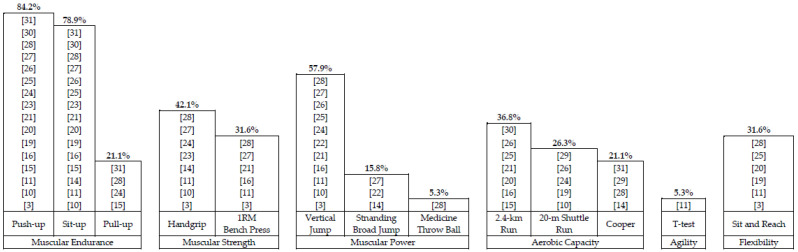
Distribution of studies [3,10,11,14,15,16,19,20,21,22,23,24,25,26,27,28,29,30,31] in each fitness test proposed by Massuça et al. [9].

The most used fitness components were in muscular endurance, the push-up, sit-up, and pull-up tests used in seventeen studies [3,10,11,14,15,16,19,20,21,23,24,25,26,27,28,30,31]. The handgrip test and 1 RM bench press were used for muscular strength in ten studies [3,10,11,14,16,21,23,24,27,28]. For muscle power, the vertical jump, standing broad jump, and medicine ball throw were the main tests used in twelve studies [3,10,11,14,16,21,22,24,25,26,27,28]. For aerobic capacity, the most used tests were the 2.4-km (1.5-mile) run, the 20-m shuttle run, and the Cooper (12-min run), which were used in fourteen studies [10,14,15,16,19,20,21,24,25,26,28,29,30,31]. For agility, was used the classical *T*-test in one study [11], and for flexibility, the sit-and-reach test in six studies [3,11,19,20,25,28]. Figure 2 shows the main fitness tests proposed by Massuça et al. [9] for muscular endurance, strength, power, aerobic capacity, agility, and flexibility, as well as the respective studies in which they were included in the fitness assessment protocol and the percentage of their use.

In addition, it was observed that in some of the studies with participants of both sexes, the results of the fitness tests were not presented separately for males and females (i.e., the average value of joint performance is given). Table 4 identifies the studies where this is verified.

### 3.3. Meta-Analysis

**Figure 3:**
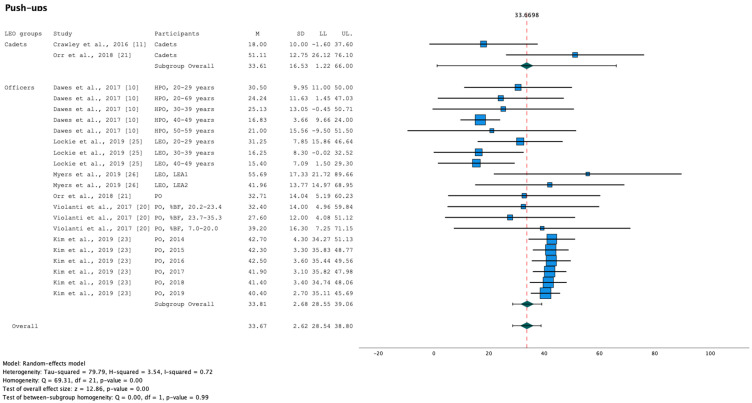
Forest plot summarising the meta-analysis results for push-ups in female LEO [10,11,20,21,23,25,26] with markers representing mean values and error bars representing 95% confidence intervals (LL and UL). Key: %BF, relative body fat; HPO, Highway Patrol Officers; LEA, Law Enforcement Agency; LEO, Law Enforcement Officers; LL, Lower limit; M, mean; PO, Police Officers; SD, standard deviation; UL, Upper limit.

**Figure 4:**
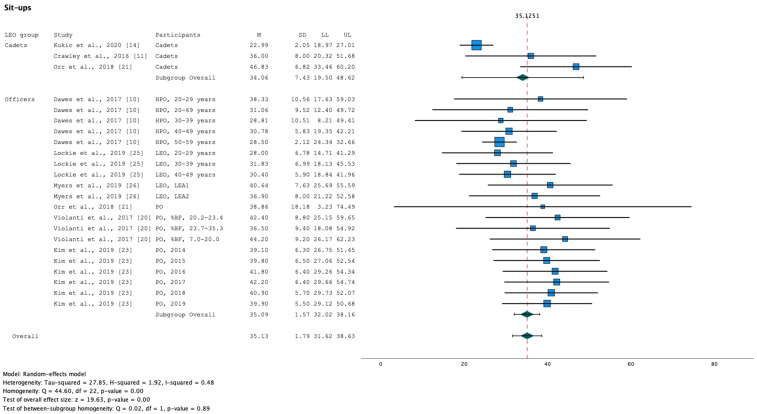
Forest plot summarising the meta-analysis results for sit-ups in female LEO [10,11,14,21,23,25,26] with markers representing mean values and error bars representing 95% confidence intervals. Key: %BF, relative body fat; HPO, Highway Patrol Officers; LEA, Law Enforcement Agency; LEO, Law Enforcement Officers; LL, Lower limit; M, mean; PO, Police Officers; SD, standard deviation; UL, Upper limit.

**Figure 5:**
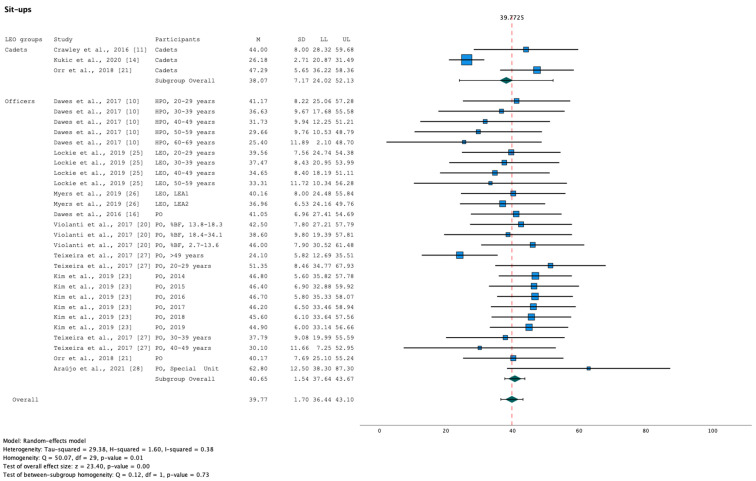
Forest plot summarising the meta-analysis results for sit-ups in male LEO [10,11,14,20,21,23,25,26,27,28] with markers representing mean values and error bars representing 95% confidence intervals. Key: %BF, relative body fat; HPO, Highway Patrol Officers; LEA, Law Enforcement Agency; LEO, Law Enforcement Officers; LL, Lower limit; M, mean; PO, Police Officers; SD, standard deviation; UL, Upper limit.

Results indicate significant heterogeneity in the female LEO results of push-ups (Q [df, 21]) = 69.31, *p* < 0.001; Figure 3) and sit-ups (Q [df, 22]) = 44.60, *p* < 0.001; Figure 4). Not only in female LEO but also in male LEO, results of the meta-analysis indicate significant heterogeneity of sit-ups (Q [df, 29]) = 50.07, *p* = 0.01; Figure 5).

The effect of LEO groups (cadets and officers) as a moderator of fitness tests was evaluated. The mixed effect model only indicates a statistically significant moderator effect in female sit-and-reach (Q_M_ [df, 1] = 9.21, *p* < 0.001), i.e., performance in push-ups, sit-ups, handgrip (dominant), 1 RM bench press, vertical jump, and 2.4-km run do not differ significantly among the LEO groups. However, small sample sizes in LEO cadets may have reduced the statistical significance of differences among samples.

Aggregation of fitness tests in male LEO based on meta-analysis, including the subgroup analysis (LEO: cadets and officers), were summarised for females in Table 5 and males in Table 6.

## 4. Discussion

This qualitative review aimed to document, compare, and examine the reference data available in the literature regarding fitness tests for LEOs. All studies showed acceptable methodological quality in the assessment of fitness attributes.

This review also provides a detailed analysis of existing data and objective reference data for essential physical skills in the components of fitness for LEO cadets and officers. One of the strengths of this study is the pioneering methodology used to establish reference data for the fitness assessment of LEOs.

Our data provide a basis for developing effective measures to improve each aspect of police officer fitness testing. The test battery includes assessments of muscular endurance, strength, power, aerobic capacity, agility, and flexibility, the essential skills for the job. The tests have acceptable technical measurement errors and high reproducibility and are assumed to be used in our environment without interference.

Physical fitness testing is a valuable tool for assessing an individual’s health status, identifying health-related risk factors, and determining job readiness and suitability.

The primary objective of physical fitness testing is to optimise functional fitness. To achieve this, it is crucial to understand the physical fitness requirements for the occupation and design or use tests that effectively measure the fitness level of recruits and officers. The results of these tests can guide exercise prescription and goal setting, which can help optimise adherence to the program, reduce injury risk, and enhance both physical and mental job performance.

It is thus evident that the need to profile fitness tests for LEOs can improve physical and overall job performance. Nevertheless, when selecting a physical assessment battery, it is essential to consider various variables, including the test population, available time, equipment and resources, and the specific information to gather from the tests.

Moreover, the standard scores obtained from fitness tests are essential for establishing health-related norms to assist individuals in setting performance goals and serve as motivational tools. Fitness tests can also positively affect individuals by fostering personal growth, reducing anxiety, and increasing motivation and confidence. Therefore, proper analysis and selection of the testing battery can help optimise the individual’s physical fitness of LEOs and positively impact their overall well-being.

According to the literature, Orr et al. [34] showed that female police officers have a moderate to strong significant relationship with all fitness measures and influence officer performance. However, the meta-analysis conducted in this study found significant heterogeneity in the results of push-ups and sit-ups among female LEOs, suggesting that there may be differences in the performance of these fitness tests among female LEOs from different populations. This variability may be attributed to several factors, including differences in physical fitness levels, variations in training programs, and cultural and social factors that may affect an individual’s level of physical activity. For example, it is hypothesised that female LEOs may face physical activity and fitness barriers due to workplace sexism and the lack of peer and supervisor support. Also, employment in a non-traditional occupation, like female LEOs, where males often deliver training, can be a reason for this disparity because males and females may approach task performance differently. On the other hand, there were no significant differences in the performance of push-ups, sit-ups, handgrip (dominant), 1 RM bench press, vertical jump (Sargent/Abalakov), and 2.4-km run between LEO cadets and officers, suggesting that training level or experience did not significantly affect the performance of these fitness tests.

The proposal to develop a battery of fitness tests stems from the need to assess and diagnose LEO’s physical fitness. Given the physical demands of the police profession, specific assessment tests and the development of norm tables are needed to verify the relevance of these assessment results. The normative reference approach is used to evaluate the performance of the incumbent and officials against a normative sample, and a statistical procedure is used to establish a standard. However, a critical step in conducting a fitness test is establishing a minimally acceptable standard. It is important to note that standard setting should be reasonable and involves complex legal considerations. To ensure that standards are reliable and valid, professionals with relevant expertise should be involved in setting the standards. They can use various methods, such as job analyses and evidence-based research, to establish appropriate standards. When developing the standards, it is also essential to consider the tested people’s specific job requirements and characteristics. This also applies to the presentation of results. As expected, the number of tests and the reported outcome variables show significant variability in how the fitness attributes of LEOs are tested. Although many personal factors can influence the results of a fitness assessment, this study attempted to account for unique characteristics to obtain homogeneous samples. In addition, most studies show heterogeneity between protocols used to measure components of fitness or the same protocol when results are presented for police populations. Therefore, comparing results between studies is difficult due to differences in assessment methods.

The second main objective of this qualitative review was to establish reference values for the main fitness tests adapted for LEO. Nevertheless, comparing the normative means of the studies raises some questions about the methodology, applicability, and presentation of the results. In other words, some literature provided preliminary results and had several limitations, such as the fact that some authors presented male and female average values of fitness assessments together [15,19,24,26,29,30,31], others did not use the same units of measurement, and some authors presented few results or differentiated according to different age groups, which made the definition of reference values very difficult.

The meta-analysis showed heterogeneity in some fitness test results among LEOs groups, possibly due to differences in fitness levels, training programs, and cultural and social factors. The lack of homogeneity in the presentation of reference values and the lack of complete results were cited as significant limitations of the study. Since a substantial limitation of this study is the need for more homogeneity in the presentation of reference values and the absence of complete results, this work aims (complementarily) to define the scoring rules to establish and develop reference values adapted to LEOs in the future, i.e.,: (i) all tests must be performed with the same methodology and collected with the same units of measurement; (ii) the units of measurement most used were those for function according to Massuça et al. [9] (muscular endurance-all results must be reported in repetitions; muscle strength-in kg; muscle power-in centimetres for the vertical jump or in meters for medicine ball throw; aerobic capacity-in meters or minutes or maximum rate of oxygen consumption-*V*O_2_max; agility-in seconds; flexibility-in centimetres); (iii) all results must be reported by gender (males or females) and by four age groups (i.e.,: <29 years; 30–39 years; 40–49 years; >50 years). In this way, in the future, as more studies follow these criteria, we will be able to compile multiple international results and use them in a way that is more appropriate for LEOs and define reference values for setting cohort boundaries for assessment and career advancement as positive baseline values. It is suggested that further research be conducted to evaluate these criteria, as we have been able to define good cut-off points.

## 5. Conclusions

The risks associated with policing have numerous complex and long-lasting consequences that can affect the effectiveness of police operations and activities. It is critical to maintain optimal physical fitness over time, monitor changes in police officer health, and provide timely information about the positive and negative effects of irresponsible management of these issues by police officers and police management.

This qualitative review highlights the importance of optimal fitness in LEOs. It provides (i) a methodological definition for the physical fitness assessment that helps select the most used fitness tests, (ii) a standardised methodology for establishing reference data for fitness tests appropriate for LEOs; and (iii) aggregate reference values for selected fitness tests.

The battery of fitness tests should include assessments of muscular endurance, strength, power, aerobic capacity, agility, and flexibility, which are essential occupational skills. Proper classification of fitness results to establish reference values raises awareness of optimal, salient, or diminished fitness attributes in LEOs with higher scores than the general population.

In sum, our study seems to provide a basis for developing effective interventions (to improve fitness testing interpretations for LEOs) and to improve the selection and reintegration procedures (considering that this professional group performs its duties in an environment and under conditions that differ from those of other occupational groups).

## Figures and Tables

**Table 1:** Databases and Relevant Search Terms.

Databases	Search Terms	Filters (Sort by)	Results
PubMed	“Police” OR “Law enforcement” AND “Fitness test” OR “Physical fitness” AND “health”	Best Match	177
ScienceDirect	“Police” AND “Fitness test” AND “health”	Relevance	1702

**Table 2:** Databases Critical Appraisal Skill Programme (CASP) Checklist [18].

Study	Q1	Q2	Q3	Q4	Q5	Q6	Q7	Q8	Q9	Q10
Beck et al., 2015 [3]	yes	yes	yes	yes	yes	yes	yes	yes	yes	09/09
Crawley et al., 2016 [11]	yes	yes	yes	yes	yes	yes	yes	yes	yes	09/09
Dawes et al., 2016 [16]	yes	yes	yes	yes	yes	yes	yes	yes	yes	09/09
Losty et al., 2016 [19]	yes	yes	yes	yes	yes	yes	yes	yes	yes	09/09
Dawes et al., 2017 [10]	yes	yes	yes	yes	yes	yes	yes	yes	yes	09/09
Violanti et al., 2017 [20]	yes	yes	yes	yes	yes	yes	yes	yes	yes	09/09
Orr et al., 2018 [21]	yes	yes	yes	yes	yes	yes	yes	yes	yes	09/09
Frio Marins et al., 2019 [22]	yes	yes	yes	yes	yes	yes	yes	no	yes	08/09
Kim et al., 2019 [23]	yes	yes	yes	yes	yes	yes	yes	no	yes	08/09
Lentz et al., 2019 [24]	yes	yes	yes	yes	yes	yes	yes	yes	yes	09/09
Lockie et al., 2019 [25]	yes	yes	yes	yes	yes	yes	yes	yes	yes	09/09
Myers et al., 2019 [26]	yes	yes	yes	yes	yes	yes	yes	no	yes	08/09
Teixeira et al., 2019 [27]	yes	yes	yes	yes	yes	yes	yes	yes	yes	09/09
Kukić et al., 2020 [14]	yes	yes	yes	yes	yes	yes	yes	yes	yes	09/09
Lockie et al., 2020 [15]	yes	yes	yes	yes	yes	yes	yes	yes	yes	09/09
Araújo et al., 2021 [28]	yes	yes	yes	yes	yes	yes	yes	yes	yes	09/09
Caetano et al., 2021 [29]	yes	yes	yes	yes	yes	yes	yes	no	yes	08/09
Lockie et al., 2021 [30]	yes	yes	yes	yes	yes	yes	yes	yes	yes	09/09
Sá et al., 2021 [31]	yes	yes	yes	yes	yes	yes	yes	no	yes	08/09

Questions to help you make sense of Qualitative research [18]: Q1. Was there a clear statement of the aims of the research? Q2. Is a qualitative methodology appropriate? Q3. Was the research design appropriate to address the aims of the research? Q4. Was the recruitment strategy appropriate to the aims of the research? Q5. Was the data collected in Yes a way that addressed the research issue? Q6. Has the relationship between the researcher and participants been adequately considered? Q7. Have ethical issues been taken into consideration? Q8. Was the data analysis sufficiently rigorous? Q9. Is there a clear statement of findings? Q10 (Quality Score). How valuable is the research?

**Table 4:** Fitness tests included in the fitness protocol and the sex of participants in each study.

Study	Muscular Endurance			Muscular Strength		Muscular Power			Aerobic Capacity			Agility	Flexibility
	Push-Ups	Sit-Ups	Pull-Ups	Handgrip	1 RM Bench Press	Vertical Jump	Standing Broad Jump	Medicine Throw Ball	20-m Shuttle Run	2.4-km Run	Cooper	*t*-Test	Sit-and-Reach
Beck et al., 2015 [3]	♂			♂	♂	♂							♂
Crawley et al., 2016 [11]	♀♂, ♀, ♂	♀♂, ♀, ♂		♀♂, ♀, ♂	♀♂, ♀, ♂	♀♂, ♀, ♂						♀♂, ♀, ♂	♀♂, ♀, ♂
Dawes et al., 2016 [16]	♂	♂			♂	♂				♂			
Losty et al., 2016 [19]	♀♂	♀♂							♀♂				♀♂
Dawes et al., 2017 [10]	♀♂, ♀, ♂	♀♂, ♀, ♂		♀♂, ♀, ♂		♀♂, ♀, ♂			♀♂, ♀, ♂				
Violanti et al., 2017 [20]	♀, ♂	♀, ♂								♀, ♂			♀, ♂
Orr et al., 2018 [21]	♀, ♂	♀, ♂			♀, ♂	♀, ♂				♀, ♂			
Frio Marins et al., 2019 [22]						♂	♂						
Kim et al., 2019 [23]	♀, ♂	♀, ♂		♀, ♂									
Lentz et al., 2019 [24]	♀♂		♀♂	♀♂		♀♂			♀♂				
Lockie et al., 2019 [25]	♀, ♂	♀, ♂				♀, ♂				♀, ♂			♀, ♂
Myers et al., 2019 [26]	♀♂, ♂	♀♂, ♂				♀♂, ♂			♀♂	♀♂			
Teixeira et al., 2019 [27]	♂	♂		♂	♂	♂	♂						
Kukić et al., 2020 [14]		♀♂, ♀, ♂		♀♂, ♀, ♂			♀♂, ♀, ♂				♀♂, ♀, ♂		
Lockie et al., 2020 [15]	♀♂	♀♂	♀♂							♀♂			
Araújo et al., 2021 [28]	♂	♂	♂	♂	♂	♂		♂			♂		♂
Caetano et al., 2021 [29]	(♀♂ unclear)		(♀♂ unclear)						♀♂		♀♂		
Lockie et al., 2021 [30]	♀♂	♀♂								♀♂			
Sá et al., 2021 [31]	♀♂	♀♂	♀♂								♀♂		

*Key: ♀♂, male plus female; ♀, female; ♂, male.*

**Table 5:** Aggregation of fitness tests in female LEO based on meta-analysis, including the subgroup analysis (LEO: cadets and officers).

Physical Capacity	Fitness Tests	Groups	Studies	n	Mean	SD	Z	*p*-Value	95% Confidence Interval		Meta-Analysis (Cochran’s Q-Statistic)
									Lower	Upper	
Muscular Endurance	Push-ups (repetitions)	Cadets	[11,21]	24	33.61	16.53	2.034	<0.001	1.22	66.00	Q [df, 21] = 69.31, *p* < 0.001; I^2^ = 0.72, τ^2^ = 79.79 Q_M_ [df, 1] = 0.00, *p* = 0.99
		Officers	[10,20,21,23,25,26]	226	33.81	2.68	12.605	<0.001	28.55	39.06	
		Overall	[10,11,20,21,23,25,26]	250	33.67	2.62	12.856	<0.001	28.54	38.80	
	Sit-ups (repetitions)	Cadets	[11,14,21]	103	34.06	7.43	4.585	<0.001	19.50	48.62	Q [df, 22] = 44.60, *p* < 0.001; I^2^ = 0.48, τ^2^ = 27.85 Q_M_ [df, 1] = 0.02, *p* = 0.89
		Officers	[10,20,21,23,25,26]	226	35.09	1.57	22.392	<0.001	32.02	38.16	
		Overall	[10,11,14,20,21,23,25,26]	329	35.13	1.79	19.627	<0.001	31.62	38.63	
Muscular Strength	Handgrip (dominant) (kg)	Cadets	[14]	79	39.03	4.26	9.162	<0.001	30.68	47.38	Q [df, 5] = 4.71, *p* = 0.45; I^2^ = 0.08, τ^2^ = 2.02 Q_M_ [df, 1] = 0.04, *p* = 0.84
		Officers	[10]	34	40.01	2.49	16.048	<0.001	35.12	44.89	
		Overall	[10,14]	113	39.89	2.03	19.688	<0.001	35.92	43.87	
	1 RM bench press (kg)	Cadets	[11,21]	24	44.64	9.36	4.770	<0.001	26.30	62.98	Q [df, 2] = 1.59, *p* = 0.45; I^2^ = 0.00, τ^2^ = 0.00 Q_M_ [df, 1] = 0.00, *p* = 0.94
		Officers	[21]	7	45.45	6.82	6.660	<0.001	32.08	58.82	
		Overall	[11,21]	31	44.21	4.86	9.090	<0.001	34.67	53.74	
Muscular Power	Vertical jump (Sargent/Abalakov) (cm)	Cadets	[11,21]	24	42.86	3.25	13.185	<0.001	36.49	49.23	Q [df, 12] = 19.22, *p* = 0.08; I^2^ = 0.34, τ^2^ = 16.32 Q_M_ [df, 1] = 0.51, *p* = 0.48
		Officers	[10,21,25,26]	73	39.96	2.46	16.251	<0.001	35.14	44.78	
		Overall	[10,11,21,25,26]	97	40.39	1.99	20.331	<0.001	36.50	44.29	
Aerobic Capacity	2.4-km (1.5-mile) run (min)	Cadets	[21]	18	12.35	0.82	15.061	<0.001	10.74	13.96	Q [df, 7] = 11.15, *p* = 0.13; I^2^ = 0.30, τ^2^ = 1.04 Q_M_ [df, 1] = 2.54, *p* = 0.11
		Officers	[20,21,25]	143	14.19	0.81	17.522	<0.001	12.60	15.77	
		Overall	[20,21,25]	161	13.67	0.68	20.164	<0.001	12.35	15.00	
Flexibility	Sit-and-reach (cm)	Cadets	[11]	6	32.10	6.20	5.177	<0.001	19.95	44.25	Q [df, 6] = 10.22, *p* = 0.12; I^2^ = 0.44, τ^2^ = 30.00 Q_M_ [df, 1] = 9.71, *p* < 0.001
		Officers	[20,25]	136	52.87	2.44	21.681	<0.001	48.09	57.64	
		Overall	[11,20,25]	142	49.47	3.20	15.465	<0.001	43.20	55.74	

Key: I^2^, percentage of variability in effect sizes which is not due to sampling error; Q, Cochran’s Q-statistic (weighted sum of squares); Q_M_, Cochran’s Q-statistic for subgroups; SD, standard deviation; τ^2^, between-study variance in each set of samples.

**Table 6:** Aggregation of fitness tests in male LEO based on meta-analysis, including the subgroup analysis (LEO: cadets and officers).

Physical Capacity	Fitness Tests	Groups	Studies	n	Mean	SD	Z	*p*-Value	95% Confidence Interval		Meta-Analysis (Cochran’s Q-Statistic)
									Lower	Upper	
Muscular Endurance	Push-ups (repetitions)	Cadets	[11,21]	115	59.02	11.61	5.082	<0.001	36.26	81.78	Q [df, 29] = 18.77, *p* = 0.93; I^2^ = 0.00, τ^2^ = 00.00 Q_M_ [df, 1] = 2.24, *p* = 0.13
		Officers	[3,10,16,20,21,23,25,26,27]	3768	41.37	1.99	20.791	<0.001	37.47	45.27	
		Overall	[3,10,11,16,20,21,23,25,26,27]	3883	42.22	1.94	21.739	<0.001	38.41	46.03	
	Sit-ups (repetitions)	Cadets	[11,14,21]	213	38.07	7.17	5.309	<0.001	24.02	52.13	Q [df, 29] = 50.07, *p* = 0.01; I^2^ = 0.38, τ^2^ = 29.38 Q_M_ [df, 1] = 0.12, *p* = 0.73
		Officers	[10,16,20,21,23,25,26,27,28]	3869	40.65	1.54	26.429	<0.001	37.64	43.67	
		Overall	[10,11,14,16,20,21,23,25,26,27,28]	4082	39.77	1.70	23.405	<0.001	36.44	43.10	
Muscular Strength	Handgrip (dominant) (kg)	Cadets	[14]	98	63.19	7.24	8.728	<0.001	49.00	77.38	Q [df, 5] = 2.98, *p* = 0.70; I^2^ = 0.00, τ^2^ = 0.00 Q_M_ [df, 1] = 2.14, *p* = 0.14
		Officers	[10]	597	51.99	2.49	20.863	<0.001	47.11	56.88	
		Overall	[10,14]	695	53.18	2.36	22.568	<0.001	48.56	57.80	
	1 RM bench press (kg)	Cadets	[11,21]	115	97.18	17.09	5.687	<0.001	63.69	130.67	Q [df, 9] = 9.16, *p* = 0.42; I^2^ = 0.37, τ^2^ = 181.98 Q_M_ [df, 1] = 0.51, *p* = 0.47
		Officers	[3,16,21,27,28]	379	83.79	7.68	10.913	<0.001	68.75	98.84	
		Overall	[3,11,16,21,27,28]	494	86.12	7.42	11.614	<0.001	71.59	100.65	
Muscular Power	Vertical jump (Sargent/Abalakov) (cm)	Cadets	[11,21]	115	61.43	6.76	9.093	<0.001	48.19	74.67	Q [df, 15] = 18.18, *p* = 0.25; I^2^ = 0.29, τ^2^ = 23.24 Q_M_ [df, 1] = 1.43, *p* = 0.23
		Officers	[3,10,16,21,25,26]	1511	52.86	2.40	22.017	<0.001	48.15	57.56	
		Overall	[3,10,11,16,21,25,26]	1626	53.62	2.28	23.483	<0.001	49.14	58.09	
Aerobic Capacity	2.4-km (1.5-mile) run (min)	Cadets	[21]	66	11.01	1.17	9.410	<0.001	8.72	13.30	Q [df, 9] = 6.39, *p* = 0.70; I^2^ = 0.00, τ^2^ = 0.00 Q_M_ [df, 1] = 1.57, *p* = 0.21
		Officers	[16,20,21,25]	2337	12.68	0.65	19.593	<0.001	11.41	13.95	
		Overall	[16,20,21,25]	2403	12.29	0.57	21.699	<0.001	11.18	13.40	
Flexibility	Sit-and-reach (cm)	Cadets	[11]	49	28.00	8.50	3.294	<0.001	11.34	44.66	Q [df, 9] = 8.36, *p* = 0.50; I^2^ = 0.00, τ^2^ = 0.00 Q_M_ [df, 1] = 3.07, *p* = 0.08
		Officers	[3,20,25,28]	2321	43.56	2.57	16.966	<0.001	38.52	48.59	
		Overall	[3,11,20,25,28]	2370	42.25	2.46	17.193	<0.001	37.44	47.07	

Key: I^2^, percentage of variability in effect sizes which is not due to sampling error; Q, Cochran’s Q-statistic (weighted sum of squares); Q_M_, Cochran’s Q-statistic for subgroups; SD, standard deviation; τ^2^, between-study variance in each set of samples.

## Data Availability

Not applicable.

## References

[1] Stanish H.I., Wood T.M., Campagna P. (1999). Prediction of performance on the RCMP physical ability requirement evaluation. J. Occup. Environ. Med..

[2] Strating M., Bakker R.H., Dijkstra G.J., Lemmink K.A., Groothoff J.W. (2010). A job-related fitness test for the Dutch police. Occup. Med..

[3] Beck A.Q., Clasey J.L., Yates J.W., Koebke N.C., Palmer T.G., Abel M.G. (2015). Relationship of Physical Fitness Measures vs. Occupational Physical Ability in Campus Law Enforcement Officers. J. Strength Cond. Res..

[4] Lockie R.G., Balfany K., Bloodgood A.M., Moreno M.R., Cesario K.A., Dulla J.M., Dawes J.J., Orr R.M. (2019). The Influence of Physical Fitness on Reasons for Academy Separation in Law Enforcement Recruits. Int. J. Environ. Res. Public Health.

[5] Shusko M., Benedetti L., Korre M., Eshleman E.J., Farioli A., Christophi C.A., Kales S.N. (2017). Recruit Fitness as a Predictor of Police Academy Graduation. Occup. Med..

[6] Dawes J., Lockie R.G., Orr R.M., Kornhauser C., Holmes R. (2019). Initial Fitness Testing Scores as a Predictor of Police Academy Graduation. J. Aust. Strength Cond..

[7] Petersen S.R., Anderson G.S., Tipton M.J., Docherty D., Graham T.E., Sharkey B.J., Taylor N.A. (2016). Towards best practice in physical and physiological employment standards. Appl. Physiol. Nutr. Metab..

[8] Mantilla-Rodríguez J.P., Hernández-Cortés P.L., Enríquez-Reyna M.-C., Carranza-Garcia L.E. (2021). Proposal of normative values for the physical evaluation of police officers. J. Hum. Sport Exerc..

[9] Massuça L.M., Santos V., Monteiro L.F. (2022). Identifying the Physical Fitness and Health Evaluations for Police Officers: Brief Systematic Review with an Emphasis on the Portuguese Research. Biology.

[10] Dawes J.J., Orr R.M., Flores R.R., Lockie R.G., Kornhauser C., Holmes R. (2017). A physical fitness profile of state highway patrol officers by gender and age. Ann. Occup. Environ. Med..

[11] Crawley A.A., Sherman R.A., Crawley W.R., Cosio-Lima L.M. (2016). Physical Fitness of Police Academy Cadets: Baseline Characteristics and Changes During a 16-Week Academy. J. Strength Cond. Res..

[12] Yoo H.L., Franke W.D. (2009). Prevalence of cardiovascular disease risk factors in volunteer firefighters. J. Occup. Environ. Med..

[13] Adams J., Cheng D., Lee J., Shock T., Kennedy K., Pate S. (2014). Use of the bootstrap method to develop a physical fitness test for public safety officers who serve as both police officers and firefighters. Bayl. Univ. Med. Cent. Proc..

[14] Kukić F., Lockie R.G., Vesković A., Petrović N., Subošić D., Spasić D., Paspalj D., Vulin L., Koropanovski N. (2020). Perceived and Measured Physical Fitness of Police Students. Int. J. Environ. Res. Public Health.

[15] Lockie R.G., Dawes J.J., Orr R.M., Dulla J.M. (2020). Recruit Fitness Standards From a Large Law Enforcement Agency: Between-Class Comparisons, Percentile Rankings, and Implications for Physical Training. J. Strength Cond. Res..

[16] Dawes J.J., Orr R.M., Siekaniec C.L., Vanderwoude A.A., Pope R. (2016). Associations between anthropometric characteristics and physical performance in male law enforcement officers: A retrospective cohort study. Ann. Occup. Environ. Med..

[17] Page M.J., McKenzie J.E., Bossuyt P.M., Boutron I., Hoffmann T.C., Mulrow C.D., Shamseer L., Tetzlaff J.M., Akl E.A., Brennan S.E. (2021). The PRISMA 2020 statement: An updated guideline for reporting systematic reviews. BMJ.

[18] Buccheri R.K., Sharifi C. (2017). Critical Appraisal Tools and Reporting Guidelines for Evidence-Based Practice. Worldviews Evid. Based Nurs..

[19] Losty C., Williams E., Gossman P. (2017). Police officer physical fitness to work: A case for health and fitness training. J. Hum. Sport Exerc..

[20] Violanti J.M., Ma C.C., Fekedulegn D., Andrew M.E., Gu J.K., Hartley T.A., Charles L.E., Burchfiel C.M. (2017). Associations Between Body Fat Percentage and Fitness among Police Officers: A Statewide Study. Saf. Health Work.

[21] Orr R.M., Dawes J.J., Pope R., Terry J. (2018). Assessing Differences in Anthropometric and Fitness Characteristics Between Police Academy Cadets and Incumbent Officers. J. Strength Cond. Res..

[22] Frio Marins E., Cabistany L., Bartel C., Dawes J.J., Boscolo Del Vecchio F. (2019). Aerobic fitness, upper-body strength and agility predict performance on an occupational physical ability test among police officers while wearing personal protective equipment. J. Sports Med. Phys. Fit..

[23] Kim S., Kim J. (2019). A Comparative Analysis of Physical Fitness in Korean Police Officers: Focus on Results between 2014 to 2019. Exerc. Sci..

[24] Lentz L., Randall J.R., Guptill C.A., Gross D.P., Senthilselvan A., Voaklander D. (2019). The Association Between Fitness Test Scores and Musculoskeletal Injury in Police Officers. Int. J. Environ. Res. Public Health.

[25] Lockie R.G., Dawes J.J., Kornhauser C.L., Holmes R.J. (2019). Cross-Sectional and Retrospective Cohort Analysis of the Effects of Age on Flexibility, Strength Endurance, Lower-Body Power, and Aerobic Fitness in Law Enforcement Officers. J. Strength Cond. Res..

[26] Myers C.J., Orr R.M., Goad K.S., Schram B.L., Lockie R., Kornhauser C., Holmes R., Dawes J.J. (2019). Comparing levels of fitness of police Officers between two United States law enforcement agencies. Work.

[27] Teixeira J., Monteiro L.F., Silvestre R., Beckert J., Massuça L.M. (2019). Age-related influence on physical fitness and individual on-duty task performance of Portuguese male non-elite police officers. Biol. Sport.

[28] Araújo A.O., Cancela J.M., Bezerra P., Chaves C., Rodrigues L.P. (2021). Age-related influences on somatic and physical fitness of elite police agents. Retos.

[29] Caetano H.B.S., Israel-Caetano C., López-Gil J.F., Sentone R.G., Godoy K.B.S., Cavichiolli F.R., Paulo A.C. (2021). Physical fitness tests as a requirement for physical performance improvement in officers in the military police of the state of Paraná, Brazil. Rev. Bras. Med. Trab..

[30] Lockie R.G., Dawes J.J., Orr R.M., Dulla J.M. (2021). Physical fitness: Differences between initial hiring to academy in law enforcement recruits who graduate or separate from academy. Work.

[31] Sá M., Santos T., Afonso J., Gouveia E.R., Marques A. (2021). Physical fitness and anthropometrical profile for the recruits of the elite close protection unit of the Portuguese public security police. Police Pract. Res..

[32] Higgins J.P., Thompson S.G., Deeks J.J., Altman D.G. (2003). Measuring inconsistency in meta-analyses. BMJ.

[33] Jackson A.S., Sui X., O’Connor D.P., Church T.S., Lee D.C., Artero E.G., Blair S.N. (2012). Longitudinal cardiorespiratory fitness algorithms for clinical settings. Am. J. Prev. Med..

[34] Orr R.M., Kukić F., Čvorović A., Koropanovski N., Janković R., Dawes J., Lockie R. (2019). Associations between Fitness Measures and Change of Direction Speeds with and without Occupational Loads in Female Police Officers. Int. J. Environ. Res. Public Health.

